# Editorial: Highlights in Alzheimer's and Parkinson's disease

**DOI:** 10.3389/fnhum.2023.1238525

**Published:** 2023-08-08

**Authors:** Mariagiovanna Cantone, Leonardo Sacco

**Affiliations:** ^1^Neurology Unit, Policlinico University Hospital “G. Rodolico-San Marco”, Catania, Italy; ^2^Neurocenter of Southern Switzerland, Neuropsychological and Speech Therapy Unit, Ente Cantonale Ospedaliero, Lugano, Switzerland

**Keywords:** dementia, Alzheimer's disease, Parkinson' s disease, neurodegeneration, aging

Neurodegenerative diseases are aging-related disorders characterized by a progressive decline of cognitive functions, movement disorders, and behavioral or personality changes. The prevalence of these conditions increases exponentially with the continuous growth of the elderly population. The broader individual, societal, and economic impact of Alzheimer's disease (AD) and Parkinson's disease (PD), which are recognized as the most common neurodegenerative diseases globally, implicates a huge economic burden on healthcare systems and a severe effect on the quality of life of patients, caregivers, and their families (Erkkinen et al., 2018). AD is the most common cause of primary dementia. Intracellular neurofibrillary tangles, deposits of tau protein, and extracellular beta-amyloid in the medial temporal lobe, entorhinal, and cingulate cortices, as well as in posterior cortical associative areas, are the hallmark of the disease (Soria Lopez et al., 2019). Despite the progressive impairment of associative cortical areas, neurophysiological studies have demonstrated that, in patients with AD, cortical excitability is enhanced as a compensatory mechanism to execute voluntary movements (Pennisi et al., 2011). Conversely, PD is characterized by a cardinal motor manifestation, i.e., bradykinesia, in combination with either resting tremor, rigidity, or both, as well as several equally relevant and disabling non-motor system symptoms. Among them, behavioral and personality changes and cognitive impairment leading to dementia are accepted as key non-motor features of advanced disease (Jellinger, 2012). Although the classical histological lesion in PD is the degeneration of the substantia nigra pars compacta, the thinning of the medial frontal (premotor and supplementary) motor cortex, posterior cingulate cortex, precuneus, lateral occipital and temporal cortex, as well as the dorsolateral prefrontal cortex, has been also described in the disease (Dickson, 2018).

In recent years, a large body of data has accumulated in an attempt to shed light on the pathophysiological factors underlying AD and PD. However, our understanding of these diseases is far from adequate and the treatment strategies are still not effective. This Research Topic collected both original research, opinion, methods, and perspective articles addressing innovative key aspects of the pathophysiology underlying AD, PD, and their new diagnostic and therapeutic strategies.

Various imaging and CSF biomarkers were extensively used in AD, and recently the National Institute on Aging–Alzheimer's Association (NIA-AA) Research Framework proposed to focus the definition of AD as a biological entity based on a dichotomous classification of normal (–) or abnormal biomarkers in living persons. Biomarkers are indicative of neuropathological changes and they are grouped into those of β amyloid deposition (A), pathologic tau (T), and neurodegeneration (N) termed the AT(N) biomarker grouping system (Jack et al., 2018). The AT(N) system is flexible in that new biomarkers or novel biomarkers groups can be added to the three existing AT(N) groups. The study by Rivas-Fernández et al. used structural magnetic resonance imaging (sMRI) to evaluate the volume and cortical thickness in adults with mild cognitive impairment (MCI), which may lead to differentiation among the three AT(N) biomarker-based profiles. In this context, sMRI enables the differentiation between MCI adults with and without pathological AD biomarkers. MCI represents a transitional state between healthy aging and dementia. It is characterized by a cognitive impairment that is pathological for the individual's age and education but does not meet the commonly accepted criteria for dementia. In particular, the study revealed progressive neurodegeneration along the AT(N) profiles consistent with the changing pattern observed in AD, providing new hints on the mechanisms of AD that cause cognitive decline.

The intriguing randomized sham-controlled trial by Kong et al., which analyzed the role of acupuncture as a complementary and alternative therapy in patients with mild AD, shed light on a novel, adjunctive behavioral, cognitive, and physical intervention that may improve cognition in neurodegenerative diseases, thus supporting previous findings (Lanza et al., 2018). In particular, in recent years, there has been an increasing interest in probing the effect of complementary and alternative treatments in patients with AD, such as shiatsu, which seemed to reduce depression significantly in a sample of patients with mild-to-moderate AD. Moreover, with repeated failures of single-target therapeutic treatments in the last decade, the relationship between microbial dysbiosis and several hallmarks of AD has drawn increasing attention (Liu et al., 2020). In the abovementioned study, gut microbiota was measured after acupuncture to explore the relationship between clinical efficacy and shifts of gut microbiota.

According to the suggestion by Gong et al., we anticipate that novel neurorehabilitation tools for cognitive and psychiatric symptomatology in AD will be developed in the near future. One example of a neurorehabilitation therapy is the Wuqinxi exercise, a kind of mind-body exercise that imitates the movement and breathing patterns of five animals (tigers, deer, bears, apes, and birds) and emphasizes the integration of body, breath, and mind (Guo et al., 2018). These therapies can improve clinical symptoms and induce non-invasive neuromodulation, supporting functional and structural changes in the nervous system of patients with PD and other neurodegenerative disorders.

Finally, in the overview by Goto, the specificity of the striatal dopamine D1 system in humans was examined. This article describes the effects of D1-agonists in PD patients, highlighting the D1R-mediated signaling as a key regulator of the basal ganglia functions and thus contributing to our understanding of the symptoms and therapies for basal ganglia-related movement disorders. The authors concluded that advances in pharmacological or genetic-based treatments should be encouraged to ameliorate disease burden and its progression, although caution is always recommended when interpreting genetic variants in PD, as recently highlighted by Cali et al. (2019).

In conclusion, although more extensive studies are needed, the contributions to this Research Topic may expand the knowledge of the pathological process involved in neurodegeneration by presenting different phenotypes for diagnostic refinement or by highlighting current challenges and potential therapeutic measures.

## Author contributions

MC and LS drafted the manuscript. MC conducted the analysis of data. LS revised the manuscript critically for important intellectual content. All authors approved the version of the manuscript to be published.
